# Recurrent papillary craniopharyngioma with BRAFV600E mutation treated with neoadjuvant-targeted therapy

**DOI:** 10.1007/s00701-017-3311-0

**Published:** 2017-09-16

**Authors:** Elham Rostami, Petra Witt Nyström, Sylwia Libard, Johan Wikström, Olivera Casar-Borota, Olafur Gudjonsson

**Affiliations:** 1 Section of Neurosurgery, Department of Neuroscience, Uppsala University, SE-751 85 Uppsala, Sweden; 2 Department of Immunology, Genetics and Pathology, Clinical and experimental Oncology, Uppsala University, Uppsala, Sweden; 3 Skandionkliniken, Uppsala, Sweden; 4 Department of Immunology, Genetics and Pathology, Uppsala University, Uppsala, Sweden; 5 Department of Clinical Pathology, Uppsala University Hospital, Uppsala, Sweden; 6 Department of Radiology, Uppsala University, Uppsala, Sweden

**Keywords:** BRAFV600E, RAF-inhibitor, Craniopharyngioma

## Abstract

Craniopharyngiomas are histologically benign but locally aggressive tumors in the sellar region that may cause devastating neurological and endocrine deficits. They tend to recur following surgery with high morbidity; hence, postoperative radiotherapy is recommended following sub-total resection. BRAFV600E mutation is the principal oncogenic driver in the papillary variant of craniopharyngiomas. Recently, a dramatic tumor reduction has been reported in a patient with BRAFV600E mutated, multiply recurrent papillary craniopharyngioma using a combination therapy of BRAF inhibitor dabrafenib and MEK inhibitor trametinib. Here, we report on near-radical reduction of a growing residual BRAFV600E craniopharyngioma using the same neoadjuvant therapy.

## Introduction

Craniopharyngiomas are tumors that arise from the residual cells of Rathke’s pouch in the sellar or suprasellar region, with both a cystic and solid component. Although they are benign, WHO grade I, the difficulty in curing this disease makes their growth malignant in behavior with high morbidity rates. Cushing described them as “the most baffling problem which confronts the neurosurgeon” [5]. The anatomical proximity of craniopharyngiomas to the pituitary gland, optic nerve, hypothalamus, and brainstem causes their growth to generate devastating neurological deficits. Craniopharyngiomas have a bimodal age distribution with the peak at ages 5–14 and above 50 years of age [4]. There are two pathological types: the adamantinomatous and papillary craniopharyngiomas. The adamantinomatous type mainly occurs in the first 2 decade of life, while papillary craniopharyngiomas occur in older adults [7].

The current standard treatment is surgery followed by adjuvant radiation therapy. However, there is a high recurrence rate [3], with additional morbidity. Recently, it has been demonstrated that papillary craniopharyngiomas contain the BRAFV600E mutation [2]. Based on the successful outcome of BRAF inhibitors in other cancers such as cutaneous melanoma, Brastianos et al. used a combination therapy of the RAF inhibitor dabrafenib and MEK inhibitor trametinib in a patient with multiply recurrent BRAFV600E craniopharyngioma [1]. After 35 days of treatment, the tumor volume was decreased by 85%. Here, we report on a case with recurrent craniopharyngioma following surgery that showed near-radical eradication with a combination therapy of mitogen-activated protein kinase (MAPK) pathway inhibitors.

## Case report

A 65-year-old male presented with nausea and involuntary weight loss. The patient had a history of subarachnoid hemorrhage in 1993 where no aneurysm was identified, and the patient did not suffer any sequelae.

## Presentation

The patient was admitted to the hospital because of involuntary weight loss of 15 kg during a 3-month period. Malignancy investigation including CT of the thorax and the abdomen was performed and did not reveal any pathological findings, but the blood workup showed pituitary insufficiency.

**Fig. 1:**
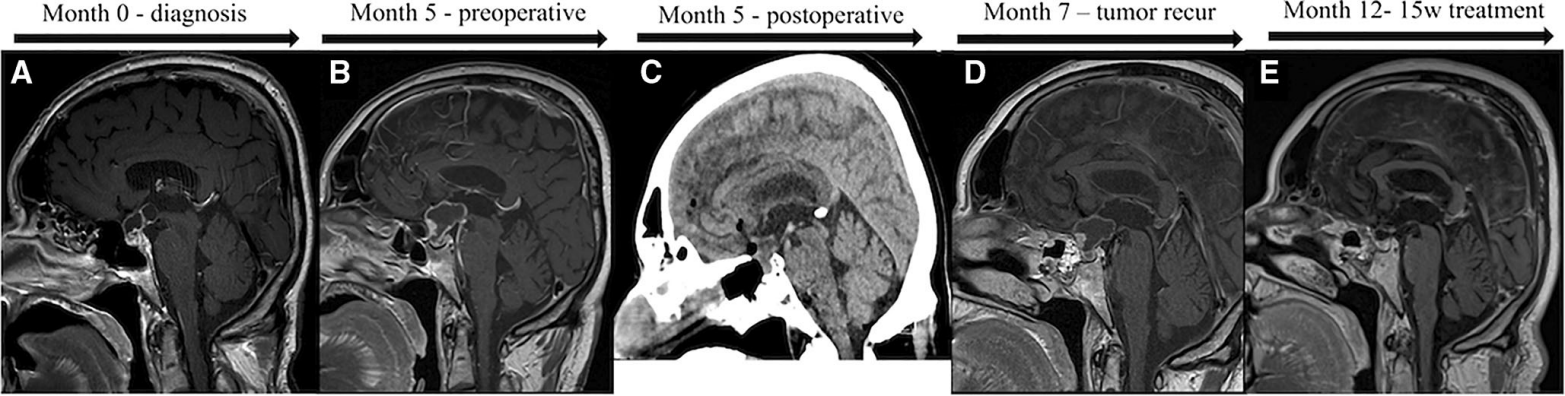
MRI images representing the time of diagnosis (**a**) until 12 months, which was the last follow-up and 15 weeks following treatment (**e**). Image **b** represents the preoperative growth, and image **c** shows the postoperative CT scan. Image **d** shows the tumor recurrence/regrowth at 2 months postoperatively

Brain CT and MRI revealed a suprasellar mass (3.1 cm^3^) (Fig. 1a) with cystic components that elevated the chiasma, and a craniopharyngioma was suspected.

Later, the patient also developed bitemporal hemianopia.

## Radiological evaluation

**Fig. 2:**
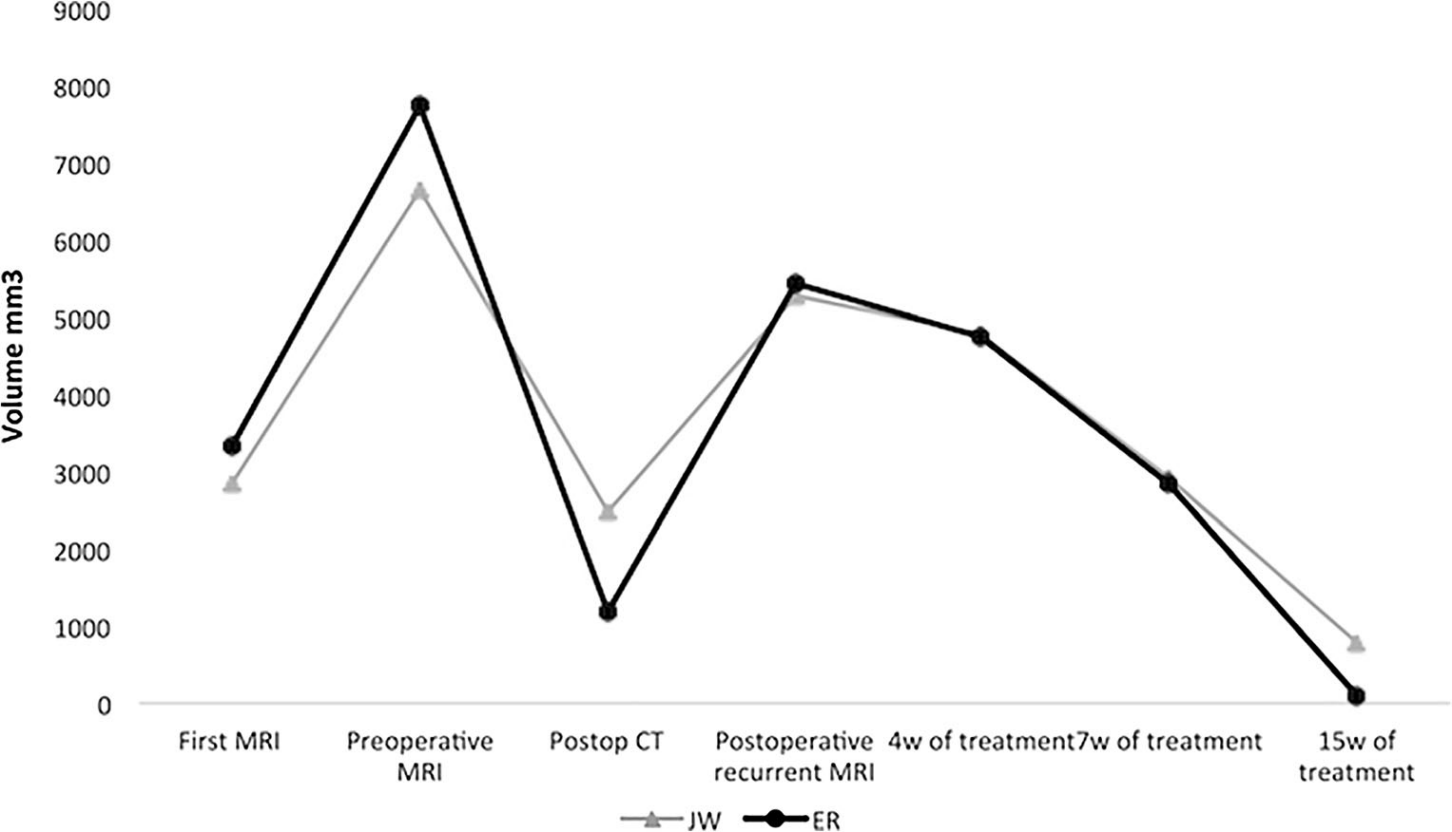
Radiological evaluation of the two readers, one neurosurgeon (ER) and one neuroradiologist (JW), who independently evaluated the CT and MRI scans. The neuroradiologist was blinded to the diagnosis and the treatment plan

Two readers, one neurosurgeon (ER) and one neuroradiologist (JW), independently evaluated the CT and MRI scans. One of the readers (JW) was blinded to the diagnosis and the treatment plan. The clinical PACS (Carestream Vue PACS, Carestream Health, Rochester, NY) was used for tumor volume measurements by manually outlining the supposed tumor border on each slice, after which the software automatically calculated the volume. The two readers’ results were quite similar and are given in Fig. 2. The below-reported values for tumor volume and volume changes represent the mean values of the two readers at each time point.

## Initial treatment

**Fig. 3:**
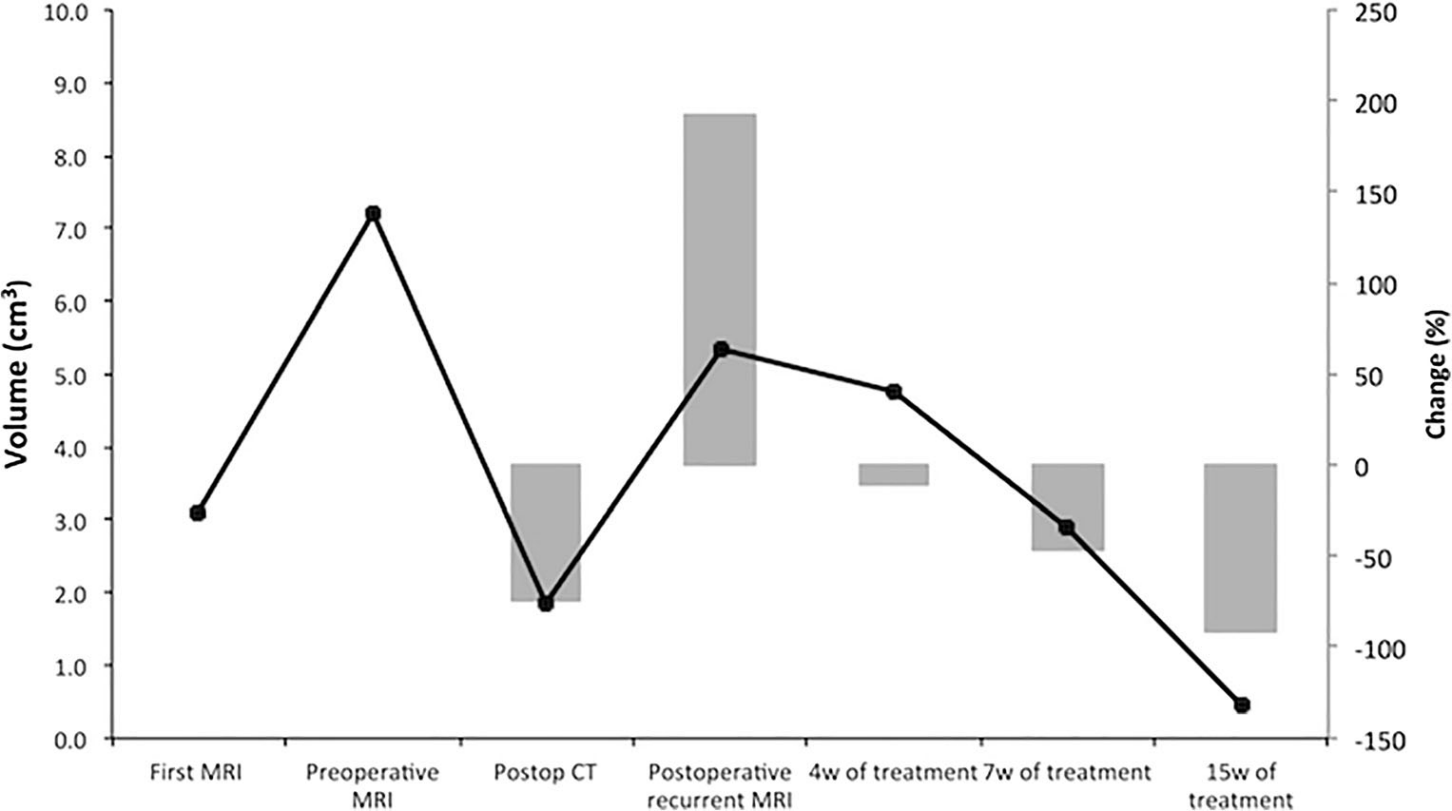
Graph illustrates the tumor volume (cm^3^) calculated as mean values of the two operators’ analysis. Bars in the graph represent the changes in the volume following the surgery (postop CT) and a combination treatment (%)

The patient was treated with replacement of glucocorticoids and thyroid hormone, and elective surgery for resection of the tumor was planned. Meanwhile, the patient’s visual deficit progressed, and a second MRI after 5 months showed a 132% increase in the tumor size (3.1 cm^3^ to 7.2 cm^3^) (Fig. 3).

The tumor and the cystic component were partially resected through a transphenoidal approach (Fig. 1c). The large cystic component of the tumor was difficult to mobilize and did not enable a total resection. Postoperatively, the patient had a significant improvement in the visual deficit.

## Histopathological and molecular genetic analysis

**Fig. 4:**
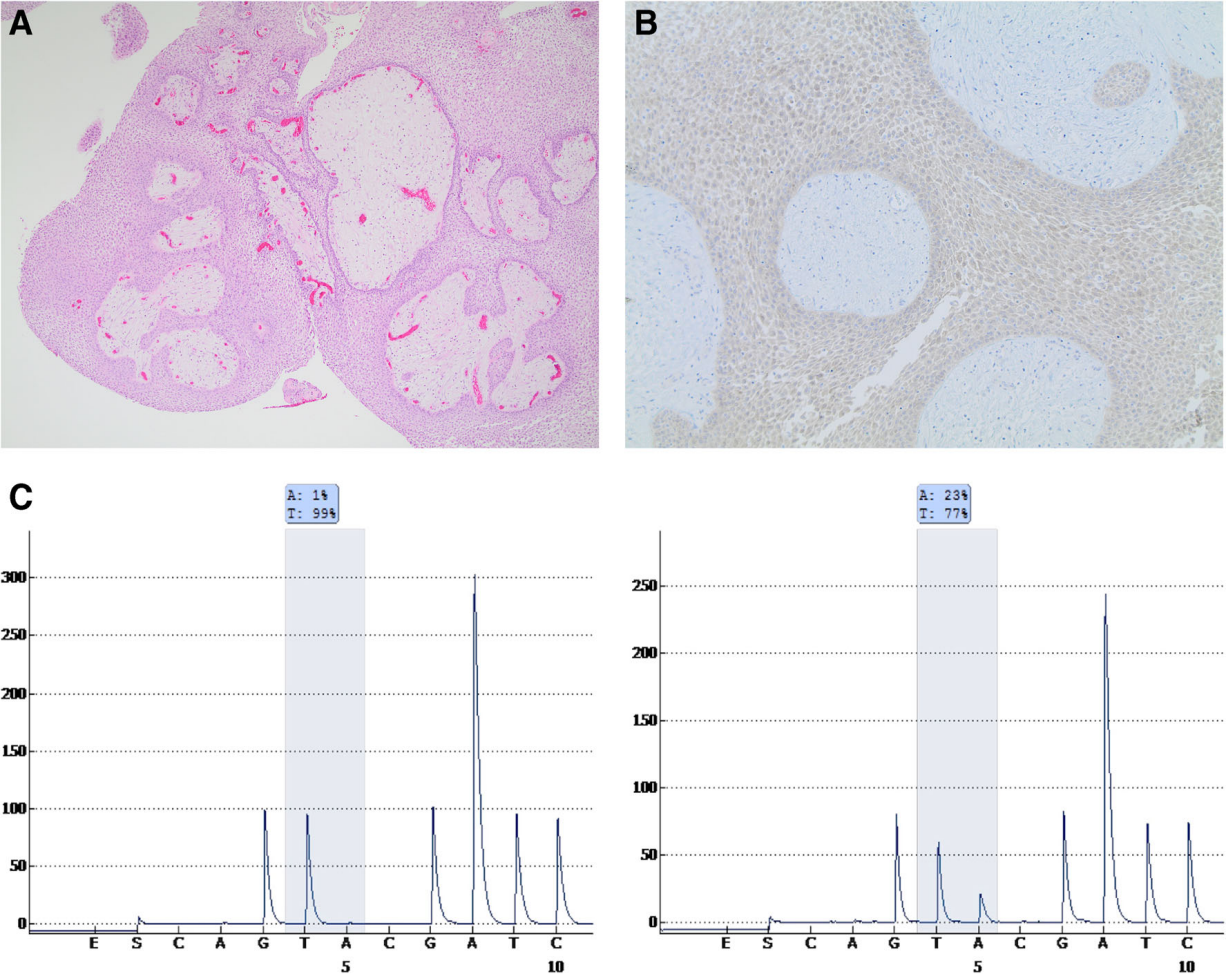
Histopathological and molecular genetic findings. Papillary craniopharyngioma. Hematoxylin-eosin staining (×40) (**a**). Immunohistochemistry, BRAFV600E (×100) (**b**). Pyrogram generated from a BRAF wild-type control specimen (**c**, left) and from the patient’s specimen demonstrating c.1799 T > A mutation resulting in the V600E mutant BRAF protein (**c**, right)

Histopathological evaluation revealed a tumor composed of fibrovascular cores lined by well-differentiated, non-keratinizing squamous epithelium, consistent with a papillary craniopharyngioma (Fig. 4a). The tumor cells demonstrated weak immunolabeling for mutated BRAFV600E protein by using immunohistochemistry [Anti-BRAFV600E (VE1) Mouse Monoclonal antibody, Ventana] (Fig. 4b). The BRAFV600E genotype was confirmed by pyrosequencing mutational analysis as previously described [8] (Fig. 4c).

## Postoperative radiological findings

Postoperative brain CT scan showed a 74% reduction of the tumor (Fig. 1), and the patient was postoperatively planned for proton radiation. During the waiting time for the radiation treatment, approximately 3 weeks, the patient again experienced visual deficits. A new brain MRI was performed and revealed regrowth of the tumor by 192% from 1.8 cm^3^ to 5.4 cm^3^ (Fig. 3).

## Postoperative treatment

Due to the fast regrowth of the tumor with a threat of vision loss, a rapid initiation of postoperative treatment was necessary. While waiting for the planning of proton radiotherapy and based on the recent success reported in the literature on BRAF inhibitors in the presence of BRAFV600E mutation in craniopharyngioma [1], the patient was treated with dabrafenib (Tafinlar) 150 mg twice daily. After 3 weeks, trametinib (Mekinist) 2 mg once daily was added, and the treatment lasted for a total of 7 weeks. Trametinib was added according to the treatment guidelines for BRAFV600E mutant melanomas.

An MRI was performed after 4 weeks of treatment, and the tumor-enhancing volume had decreased by 11%. MRI during the last week of treatment (15 weeks) showed a 91% reduction of the tumor (Fig. 3).

The patient was clinically improved with a regression of visual deficit.

Currently, the combination treatment has been paused because of drug-induced pyrexia, but will resume as soon as possible, if necessary with the support of corticosteroids. Radiotherapy will also be initiated as soon as possible.

## Discussion

This is the second case report [1] on a therapeutic response to combined BRAF and MEK-targeted therapy in a recurrent papillary craniopharyngioma with genetically confirmed BRAFV600E mutation. After 15 weeks of treatment with dabrafenib and trametinib, there was a near radical reduction of the tumor. The patient developed pyrexia, and the treatment was paused but no other side effects were detected.

Although craniopharyngiomas are benign tumors, a total resection is desirable since the rate of progression-free survival following a subtotal resection is only 34% [10]. However, a total resection is associated with high mortality and morbidity, even in the hands of skilled neurosurgeons [11]. In a systemic review analysis, it was shown that a subtotal resection with adjuvant irradiation and primary gross-total resection carry similar rates of long-term disease control and recurrence [10], and the former is currently the standard treatment. Considering the morbidity, mortality, and recurrence rate, a neoadjuvant therapy without radiation and surgery would be highly appealing.

The BRAF gene is coding for a kinase that is activated by somatic point mutation in human cancer [6]. The MAPK kinase (MEK) is downstream of BRAF in the MAPK pathway, and pharmacological inhibition of both BRAF and MEK have shown major advancements in the treatment of metastatic melanoma. However, single treatment was shown to result in progression within 6 to 7 months in 50% of patients [9]. A combination therapy with BRAF (dabrafenib) and MEK inhibitors (trametinib) showed a significantly longer progression-free survival, a higher number of patients who were alive and progression-free at 1 year, and higher tumor regression in patients with metastatic melanoma and BRAFV600E mutation [9]. In the papillary type of craniopharyngioma, it has been shown that BRAFV600E is the principal oncogenic driver [2]. Furthermore, a recent case report showed a radical tumor reduction in the papillary craniopharyngioma following a combination therapy with BRAF (dabrafenib) and MEK inhibitors (trametinib) [1].

We decided to treat our patient by using the same treatment strategy as previously described by Brastianos et al. and obtained similar results. Following 15 weeks of treatment, the tumor size was significantly reduced. Unfortunately, the patient developed pyrexia as described previously as a possible side effect [9], and the treatment was stopped.

In the case report described by Brastianos et al., BRAFV600E was also detected in the peripheral blood. Unfortunately, this sampling was not included in our study and could have a high future value if it is confirmed and proven that circulating tumor cells or cell-free DNA detected in the previous report was not a result of multiple surgical treatments.

The current finding and the previous case report indicate promising results for the postoperative treatment with BRAF (dabrafenib) and MEK inhibitors (trametinib) of BRAFV600E mutated papillary craniopharyngiomas. In the present case, this combination treatment reduced the postoperative tumor regrowth by 91%. Larger studies need to confirm and evaluate this combination treatment.
